# Cardiac Myosin Inhibitors (CMIs) and Surgical Referral in Patients with Hypertrophic Cardiomyopathy

**DOI:** 10.3390/jcdd13050187

**Published:** 2026-04-29

**Authors:** Benedetto Ferraresi, Antonio Nenna, Mohamad Jawabra, Diletta Corrado, Andrea Faggiano, Stefano Carugo, Carmelo Dominici, Giovanni Casali, Massimo Chello, Mario Lusini

**Affiliations:** 1 Cardiac Surgery, Fondazione Policlinico Universitario Campus Bio-Medico, Via Alvaro del Portillo 200, 00128 Rome, Italy; benedetto.ferraresi@unicampus.it (B.F.); mohamad.jawabra@policlinicocampus.it (M.J.); diletta.corrado@unicampus.it (D.C.);; 2 Unit of Cardiac Surgery, Department of Medicine and Surgery, Università Campus Bio-Medico di Roma, 00128 Rome, Italy; 3 Department of Cardio-Thoracic-Vascular Diseases, Foundation IRCCS Ca’ Granda Ospedale Maggiore Policlinico, 20122 Milan, Italy; antonio.nnn@hotmail.it (A.N.);; 4 Department of Clinical Sciences and Community Health, Università degli Studi di Milano, 20122 Milan, Italy; 5 Cardiac Surgery, Azienda Ospedaliero Universitaria Maggiore Della Carità di Novara, Corso Mazzini 18, 28100 Novara, Italy

**Keywords:** hypertrophic cardiomyopathy, cardiac myosin inhibitors, mavacamten, septal reduction therapy, surgical myectomy

## Abstract

The management of obstructive hypertrophic cardiomyopathy (HCM) has been transformed by the advent of cardiac myosin inhibitors (CMIs), such as mavacamten and aficamten. Unlike traditional pharmacotherapy, which primarily addresses symptoms, CMIs target the underlying mechanism of sarcomeric hypercontractility, offering significant reductions in left ventricular outflow tract (LVOT) gradients and improved functional capacity. This review evaluates the evolving role of CMIs in refining surgical candidate selection and postoperative care. Clinically, CMIs function as an in vivo “biological test” to distinguish between dynamic, functional obstruction—often manageable with medication—and fixed anatomical obstruction driven by complex septal or mitral substrates. While clinical trials demonstrate that CMIs can delay or prevent the need for SRT in a significant proportion of patients, surgery remains the definitive solution for those with dominant structural anomalies or drug intolerance. Consequently, the therapeutic paradigm is shifting from a binary “drugs or surgery” approach to a synergistic model. In this framework, CMIs optimize the identification of patients truly requiring structural myectomy while serving as a valuable adjunct for managing residual hypercontractility, ultimately facilitating a personalized, multidisciplinary approach to HCM treatment.

## 1. Introduction

Hypertrophic cardiomyopathy (HCM) is the most common inherited cardiac disease, affecting up to one in 200 adults. It is characterized by unexplained left ventricular hypertrophy and significant clinical heterogeneity, ranging from individuals who are asymptomatic to those who present with symptoms such as exertional dyspnea, angina, syncope, atrial and ventricular arrhythmias, or sudden cardiac death. The most prevalent phenotype is obstructive HCM (oHCM) when LVOT obstruction is considered either at rest (≅1/4 of patients) or provoked (up to 2/3), driven by systolic anterior motion (SAM) of the mitral valve in the setting of septal hypertrophy and mitral–subvalvular abnormalities. In contrast, non-obstructive HCM (nHCM) is primarily associated with diastolic dysfunction and microvascular ischemia, resulting in functional limitations and, in some instances, adverse outcomes [1,2,3,4]. For decades, the management of HCM has relied on treating the symptoms, such as using β-blockers, non-dihydropyridine calcium channel blockers and disopyramide in selected cases. Septal reduction therapy (SRT), in the form of surgical myectomy or alcohol septal ablation, has been used to treat patients with severe, drug-resistant obstruction. While SRT achieves excellent results in specialist centers, neither approach addresses the underlying sarcomeric hypercontractility that drives disease progression [1]. The advent of cardiac myosin inhibitors (CMIs) has transformed heart failure treatment in HCM by targeting the underlying mechanism of excessive sarcomere activation. Mavacamten, the first agent of its kind, stabilizes the super-relaxed state of myosin. It has been shown to significantly improve LVOT gradients and exercise capacity and quality of life in randomized trials. However, left ventricular ejection fraction must be carefully monitored during treatment, as there is a small risk of systolic dysfunction [2,5]. Aficamten, a next-generation CMI, has further validated this therapeutic approach by demonstrating consistent and rapid reductions in LVOT gradients. Phase 3 trials are ongoing and are summarized in Table 1. These agents are emerging as potential alternatives to, or bridges for, SRT in patients with predominantly functional obstruction. They are also valuable for refining surgical selection and identifying patients with a persistent anatomical substrate amenable to myectomy, such as marked septal hypertrophy or intrinsic mitral abnormalities [1,5]. This narrative review summarizes the current evidence on novel myosin inhibitors and explores their evolving role in selecting surgical candidates and managing patients with HCM post-operatively. This includes controlling residual hypercontractility and ventricular remodeling, as well as integrating pharmacology within a multidisciplinary ‘heart team’ framework to provide personalized care.

## 2. Rationale and Goals of Medical Therapy

The aim of medical therapy for HCM is to alleviate symptoms, reduce LVOT obstruction, improve exercise capacity and quality of life, and prevent complications such as heart failure, atrial fibrillation and sudden death. Historically, β-blockers and non-dihydropyridine calcium channel blockers (NDHP-CCBs) have been used as the primary treatment to slow heart rate, prolong diastole, and reduce dynamic obstruction [6,7]. These drugs do not alter the underlying biology of the disease; rather, they target the symptoms of sarcomeric hyperkinesia that characterize HCM, particularly in its obstructive form. In such cases, the use of cardiac myosin inhibitors (CMIs) is recommended. CMIs, such as mavacamten and aficamten, directly target sarcomeric hypercontractility by shifting myosin heads towards the super-relaxed state. This reduces the formation of actin–myosin cross-bridges and the consumption of adenosine triphosphate (ATP). These effects alleviate hypercontractility, lower LVOT gradients, and can have a favorable influence on diastolic function and reverse remodeling [8]. At the cellular level, acute exposure to mavacamten enhances diastolic compliance in cardiomyocytes. This supports the mechanistic link between cross-bridge suppression and symptom relief in oHCM, while improved lusitropy may potentially contribute to symptom improvement in phenotypes without resting obstruction [9].

## 3. Surgical Candidate Selection and Postoperative Management in the Era of Cardiac Myosin Inhibitors

In obstructive HCM, LVOT obstruction results from the interaction between sarcomeric hypercontractility and the underlying septal, mitral, and subvalvular anatomy, rather than from two fully distinct “functional” and “anatomical” mechanisms. Accordingly, treatment selection should not rely on a universal stepwise strategy in which all patients undergo a trial of cardiac myosin inhibition before consideration of septal reduction therapy. Instead, comprehensive phenotyping with resting and provoked echocardiography, complemented when appropriate by TEE or CMR, should define the dominant mechanisms of obstruction and support an informed discussion of all therapeutic options from the outset. In selected patients, CMIs may reduce gradients and symptoms and may defer invasive treatment; however, surgery remains a definitive first-line option for patients with marked septal hypertrophy, significant mitral/subvalvular abnormalities, unfavorable anatomy, drug intolerance, or a preference for one-time structural correction rather than lifelong therapy [8,10]. The first step is therefore to conduct thorough phenotyping, including resting and provoked echocardiography, as well as exercise testing where possible. Particular attention should be paid to the mitral and subvalvular apparatus using transesophageal echocardiography (TEE) and/or cardiac magnetic resonance (CMR) to detect leaflet elongation, tethering, anteriorization of the papillary muscles, accessory chordae, and other correctable anatomical abnormalities. In selected patients, CMI therapy may be initiated with echocardiography-guided titration to reduce gradients and symptoms; however, this should not be interpreted as a mandatory test before surgery. Patients with persistent symptoms or significant residual gradients despite optimized therapy, as well as those with clearly correctable septal, mitral, or subvalvular abnormalities, should be reassessed by an experienced HCM team for possible septal reduction therapy. Treatment choice should be based on anatomy, symptoms, age, comorbidities, local expertise, and patient preference [11,12]. It is also important to consider how to manage ‘partial non-responders’. Some patients treated with CMI continue to experience clinically significant symptoms or gradients, often despite unfavorable anatomical markers being present. In such cases, septal reduction therapy, particularly surgical myectomy when correctable mitral or subvalvular abnormalities are present, should be considered as a definitive treatment option. Conversely, mavacamten has demonstrated substantial reductions in gradients and improvements in symptoms in ‘difficult’ and ‘salvage’ cohorts—that is, patients who have already switched from disopyramide, pacing, aspirin or myomectomy, or who have experienced obstruction LVOTO following aortic replacement. This suggests that the drug can stabilize patients awaiting invasive treatment and prevent the need for further interventions when the residual driver is primarily functional [12,13].

## 4. Peri- and Postoperative Management in the Time of CMIs

In patients receiving CMIs, perioperative management should be integrated into a standardized pathway rather than interpreted as intraoperative drug titration. This includes preoperative assessment of LVEF and LVOT gradients, review of concomitant negative inotropic therapy such as beta-blockers and non-dihydropyridine calcium channel blockers, and postoperative reassessment before considering CMI reintroduction. Extension data show that drops in LVEF below 50% are generally reversible with temporary discontinuation followed by reintroduction at a reduced dose. However, careful echocardiogram titration is required for this process. New-onset atrial fibrillation (AF) is not uncommon in patients with HCM and requires dedicated monitoring and prevention. These principles also apply during the postoperative period when deciding whether and when to reintroduce a CMI [10]. The rationale behind the surgery is twofold. Firstly, in cases of residual dynamic obstruction following myomectomy or alcohol septal ablation (ASA), which typically involve a well-debulked septum with a marked hypercontractile component, or persistent mild MR from SAM, a CMI can ‘dampen’ the inotropic drive and encourage diastolic remodeling. Favorable outcomes have already been documented in the literature for these cases. Secondly, real-world experience shows that CMIs remain effective and well-tolerated even after surgery or ASA in patients with a history of previous advanced treatments, provided the monitoring framework (serial echocardiography, left ventricular ejection fraction (LVEF) and rhythm) is maintained [11,13].

In summary, the selection process for cardiac surgery today comprises four steps: (1) a detailed anatomical and functional assessment involving echocardiography with provocations and transesophageal echocardiography/magnetic resonance imaging targeting the mitral and papillary arteries; (2) maximum drug therapy using current treatments; (3) pharmacological testing using a CMI to assess responsiveness as a clinical biomarker of the functional component; and (4) referral for surgery if the anatomy remains dominant or the response is incomplete. Postoperatively, CMIs are used to optimize residuals and maintain remodeling within structured follow-up pathways (LVEF and gradients at recovery, β-blocker/calcium channel blocker adjustments and arrhythmia surveillance). This may reflect a shift from a simple “drug or surgery” model toward a more integrated approach, in which CMIs may help support treatment stratification in selected patients and may also have a role during structured postoperative follow-up.

## 5. Discussion

The most robust clinical trials clearly demonstrate that modulating myocardial contractility provides tangible clinical benefits for patients with hypertrophic cardiomyopathy. A pivotal moment came with the EXPLORER-HCM trial (NCT03470545), which found that mavacamten significantly improved functional capacity and symptoms, lowered the post-exercise LVOT gradient and increased peak VO_2_, while also improving quality of life, in patients with significant LVOT obstruction and preserved ejection fraction. Transient LVEF reductions were uncommon and reversible [14]. Similarly, the VALOR-HCM (NCT04349072) study showed that mavacamten can significantly reduce the need for SRT in patients, with these benefits being maintained during follow-up [14]. Taken together, these data shift therapy towards a disease-specific strategy that addresses the pathophysiology of obstruction, rather than purely providing symptomatic relief. A similar message emerges for aficamten: The SEQUOIA-HCM study, made possible by rapid titration and a broad therapeutic range, revealed a notable increase in peak VO_2_ and consistent improvements in all secondary endpoints (NYHA class, and substantial reduction in LVOT gradient), alongside a decrease in SRT eligibility, indicating the potential to postpone invasive procedures. The safety profile was comparable to placebo, with modest mean LVEF reductions and rare transient drops below 50% without clinical deterioration and full recovery after discontinuation [15]. Although potential lusitropic effects of CMIs may be relevant to nonobstructive HCM, this topic remains outside the main scope of the present review, which focuses on obstructive HCM, surgical referral, and the integration of CMIs into treatment decision-making [15,16].

Importantly, these advances unfold within a biologically heterogeneous disease in which genotype is not merely descriptive but plausibly treatment-modifying. Sarcomere-positive HCM more directly reflects primary myofilament hypercontractility—the core pharmacologic target of CMIs—whereas genotype-negative disease often represents a convergent phenotype shaped by non-sarcomeric mechanisms, comorbid remodeling, and more variable substrates of symptoms and obstruction. This heterogeneity provides a rationale for genotype-informed expectations: (i) a larger haemodynamic and symptomatic effect may be hypothesized in sarcomere-positive disease, (ii) genotype-negative patients may still respond, but with a potentially higher likelihood of ‘clinical non-response’ driven by diastolic limitation/atrial myopathy rather than LVOT physiology, and (iii) variant class across thick- vs. thin-filament pathways may contribute to inter-individual variability in efficacy and tolerability (including propensity to LVEF decline), motivating genotype-stratified analyses and real-world validation [17].

However, it is equally clear that the advent of selective cardiac myosin inhibitors (CMIs) has not rendered surgery obsolete for treating obstructive HCM. Rather, CMIs have clarified the circumstances in which surgery is necessary [10,12]. From a clinical perspective, the drug is more useful for patients who do not require it because it identifies anatomical rather than functional hypertrophy. Therefore, CMIS can play a role in identifying patients who require cardiac surgery. Until recently, patients with persistent symptoms and significant LVOT obstruction despite receiving the maximum tolerated dose of medication typically underwent SRT (surgical myectomy or alcohol septal ablation). Today, however, the pathway is more selective: a significant proportion of patients achieve clinically meaningful improvement with cardiac resynchronization therapy (CRT) and do not require immediate intervention [10,12]. In this context, CMIs target the ‘functional’ component of obstruction by reducing sarcomeric hypercontractility and the LVOT gradient. This improves NYHA class, even in patients who are already eligible for SRT [10,14]. As extended follow-up with mavacamten shows, this can delay surgery for a substantial proportion of patients with severe obstruction. Nevertheless, in many patients, obstruction is not purely dynamic but rather driven by complex anatomical substrates. In such cases, reducing contractile force alone does not normalize haemodynamics. This distinction is clinically important: some patients become haemodynamic responders (gradient reduction) but clinical non-responders (persistent dyspnoea/limited exercise capacity), particularly when advanced diastolic dysfunction, pulmonary hypertension, atrial myopathy/AF burden, or extensive fibrosis dominate symptoms. In this setting, CMR markers of fibrosis (LGE) and, when available, diffuse interstitial expansion (ECV) may help anticipate persistent limitation despite LVOT improvement and refine expectations from CMIs. Surgical myectomy therefore remains the definitive therapy when multiple structural abnormalities coexist. Surgery also retains an important role in patients with oHCM and concomitant cardiac conditions requiring operative management, including intrinsic mitral valve disease, aortic valve or ascending aortic disease, and atrial fibrillation requiring surgical ablation or left atrial appendage management. Over time, the operation has evolved into a targeted reconstructive approach involving broad septal resection combined with mitral repair to eliminate SAM and dynamic mitral regurgitation. This provides stable gradient resolution where the cause is structural [7,11,12,15]. In practice, preoperative CMI use may provide additional functional information in selected patients, although this concept still requires further validation. An echocardiography-guided titration phase integrated with rest/exercise transthoracic echocardiography, TEE and CMR allows for precise morpho-functional phenotyping. CMR complements echo by refining hypertrophy distribution and chamber geometry and by quantifying myocardial substrate (LGE/ECV), thereby strengthening the ‘CMI functional test’ paradigm to triage patients toward durable reconstructive strategies when obstruction is predominantly anatomic or symptoms are substrate-driven. If gradients, symptoms and remodeling improve significantly without intolerance (e.g., a marked drop in LVEF or new-onset AF), many patients can be managed conservatively, including those with predominantly functional obstruction who would previously have been referred for ‘default’ myectomy [7,12,15]. Conversely, persistent gradients or dyspnea despite optimal titration, or clear imaging evidence of correctable mitral or papillary apparatus abnormalities, identify the ideal surgical candidate [7,11,12]. A crucial point is that CMIs are changing the timing, rather than the necessity, of surgery. In the 128-week VALOR-HCM follow-up study, approximately 85% of patients initially referred for SRT and treated/titrated with mavacamten did not undergo the procedure, while maintaining gradient control and NYHA improvement in the medium term [10]. However, these benefits depend on continued therapy. With annual drug costs of around $80,000, a young patient who responds well to treatment could accrue around $2 million in lifetime expenses, excluding the cost of echocardiograms, clinic visits, and interruptions to treatment when the LVEF falls below 50%. Furthermore, CMIs are not curative: when treatment is stopped, obstruction often recurs, indicating predominantly symptomatic control [10]. In contrast, data from the SHARE SRT registry show a different picture: in specialist centers, periprocedural mortality is around 0.4%, 92% achieve a gradient reduction to below 50 mmHg after one year, and around 83% remain free of events after ten years—essentially a one-time solution with multi-year durability [13]. Comparative data between alcohol septal ablation and mavacamten further support the need to individualize treatment selection when balancing pharmacological therapy, percutaneous septal reduction, and surgical myectomy [18]. For a young patient with favorable anatomy (a thick septum, a prominent SAM and a correctable mitral apparatus), the real choice is not ‘drug treatment versus no treatment’, but indefinite, high-cost pharmacotherapy versus a single stabilizing procedure. This consideration is particularly relevant in younger patients, especially those who wish to avoid lifelong costly therapy, and in women planning pregnancy, in whom CMI interruption may be required. Conversely, in older patients or in those who prefer to avoid invasive treatment, CMIs may represent a reasonable definitive treatment strategy for symptomatic obstructive HCM, provided that appropriate clinical and echocardiographic monitoring is feasible. Appropriately, 2024 JACC editorials emphasize that, while CMIs are ‘revolutionary’, they should not be used as an excuse to avoid referral to HCM surgical centers. Surgery remains the only way to definitively correct structural disease, whereas drugs only keep it at bay for as long as the patient can tolerate, and afford, them [7,12,15,19]. In summary, convergent evidence from oHCM and nHCM suggests that CMIs act ‘upstream’, reducing hyperkinesis and wall stress, and producing clinically significant improvements. However, several questions remain unanswered: how to define optimal titration and monitoring (particularly for LVEF), how to identify the most responsive subgroups (including genotype/variant-informed responsiveness), and how to evaluate long-term hard outcomes and combination strategies with standard therapies. At the same time, for patients with a dominant anatomical substrate, a reconstructive surgical strategy remains indispensable. Therefore, the paradigm shift is twofold: personalization with CMIs to reduce gradients and improve functional capacity, and precise selection of candidates for SRT when obstruction is structural or refractory, to maximize both clinical benefit and cost-effectiveness [4,5,10,12,13,14].

## 6. Conclusions

Current evidence suggests that CMIs may influence the timing of surgery in patients with obstructive HCM, whereas surgical treatment remains an important definitive option when obstruction is predominantly driven by anatomical abnormalities. They are highly effective at improving mechanistic markers such as the LVOT gradient and NT-proBNP levels. However, when it comes to patient-reported outcomes such as peak VO_2_ and long-term symptom relief, the effect is more modest, and a significant proportion of improvement occurs even with placebo treatment. CMIs provide effective disease-specific symptom control while therapy is continued, but they do not directly correct fixed septal, mitral, or subvalvular abnormalities. In patients with predominantly drug-responsive obstruction, CMI therapy with structured reassessment may be appropriate. Conversely, when obstruction is driven by clearly correctable mitral, papillary muscle, or septal abnormalities, reconstructive surgical myectomy remains a definitive therapeutic option. Thus, CMIs are an important therapeutic advance, but not a universal substitute for surgery. An important potential role of CMIs may be to help refine treatment selection, including the identification of patients who may defer surgery and those who may still benefit from timely referral for septal reduction therapy. Importantly, the emergence of CMIs, particularly mavacamten, has not eliminated the role of cardiac surgery, but may contribute to a more selective and individualized referral process. Patients who now reach the operating table typically have complex structural disease, such as markedly thickened basal septum, elongated or anteriorized mitral apparatus, displaced papillary muscles, and severe SAM with dynamic mitral regurgitation [20,21]. These conditions require multilevel structural correction, which cannot be achieved through the pharmacological modulation of contractility [7,12]. In such cases, extended myectomy, often combined with mitral valve remodeling and concomitant management of atrial arrhythmias, remains the definitive treatment to abolish the outflow gradient and normalize haemodynamics [22]. This integration also reshapes follow-up. Therefore, a shared surgeon–cardiologist model incorporating serial imaging, early rhythm surveillance, targeted left atrial management and selective CMI use to modulate residual hypercontractility or diastolic stiffness is essential. In high-volume registries, surgical septal reduction demonstrates very low perioperative mortality and long-lasting symptomatic improvement, while CMIs are associated with favorable reverse remodeling of the left ventricle and left atrium [5,9,10,16,17,20]. In summary, CMIs and surgery should currently be viewed as complementary strategies within a multidisciplinary framework, with treatment choice guided by symptoms, anatomical substrate, response to therapy, and patient characteristics.

## Figures and Tables

**Table 1:** Current update of clinical trials about cardiac myosin inhibitors (CMI).

NCT Number	Study Title	Locations	Status	Inclusion Criteria	Exclusion Criteria	Study Type	Results
NCT06947590	Efficacy of Mavacamten in Patients With Symptomatic Latent Obstructive Hypertrophic Cardiomyopathy	China	Active, not recruiting	Age ≥ 18 years Weight > 45 kg Adequate acoustic windows to allow accurate transthoracic echocardiograms (TTEs) Diagnosis of latent obstructive hypertrophic cardiomyopathy according to ACCF/AHA, ESC, and Chinese Society of Cardiology guidelines Left ventricular ejection fraction (LVEF) ≥ 55% at rest, confirmed by the echocardiography core laboratory New York Heart Association (NYHA) Class II or III symptoms at screening Resting oxygen saturation ≥ 90% at screening	Any acute or severe comorbidities (e.g., severe infections; hematologic, renal, metabolic, gastrointestinal, or endocrine dysfunction) Currently using or having used prohibited medications within 14 days prior to screening (CYP2C19 inhibitors such as omeprazole/esomeprazole; strong CYP3A4 inhibitors) Life expectancy < 1 year Pregnant or breastfeeding women History of syncope or sustained ventricular tachyarrhythmia during exercise within the past 6 months Atrial fibrillation (AF) Currently receiving or planning to receive disopyramide, cibenzoline, ranolazine, or a combination of beta-blockers with verapamil or diltiazem	Medical treatment	No results posted
NCT06338202	Real-World Effectiveness of Mavacamten in Canada	Canada	Recruiting	Patients who have initiated mavacamten, as part of routine clinical care, through the Bristol Myers Squibb CAMZYOS Patient Support Program for the treatment of symptomatic obstructive hypertrophic cardiomyopathy (oHCM) Adult patient (≥18 years of age, or as defined per local province/territory) at Index Date (first prescribed dose of mavacamten) Patient has been diagnosed with symptomatic oHCM of New York Heart Association (NYHA) class II to III at the time of mavacamten initiation	In the 6 weeks prior to baseline (Index Date; first prescribed dose of mavacamten), the patient has received any cardiac myosin inhibitor (including mavacamten) for oHCM or for any other medical condition as part of a clinical trial	Observational	No results posted
NCT06023186	Effect of Mavacamten Treatment on Coronary Flow Reserve in oHCM	USA	Recruiting	Provides informed consent; willing to follow visits/procedures Age 18–85 oHCM diagnosis: LV wall ≥ 15 mm (≥13 mm if relative/genotype+), not due to other causes Prescribed mavacamten per US label Intends to adhere to oral therapy for study duration Women of childbearing potential: negative pregnancy test at baseline & 12 months	Pregnancy or lactation Known hypersensitivity to components of mavacamten or regadenoson Prior treatment with mavacamten or aficamten	Observational	No results posted
NCT05414175	A Study of Mavacamten in Obstructive Hypertrophic Cardiomyopathy	Japan	Active, not recruiting	Age ≥ 18; weight ≥ 35 kg Adequate TTE windows oHCM per ACCF/AHA, ESC, JCS guidelines LVEF ≥ 60%; NYHA II–III	Hypertrophy mimics Syncope or exercise-induced sustained VT ≤ 6 mo Resuscitated SCA (any time) or appropriate ICD shock ≤ 6 mo AF: paroxysmal with AF at screening; or persistent/permanent without ≥4 wk anticoagulation and/or not rate-controlled ≤ 6 mo Cibenzoline, disopyramide, ranolazine ≤ 14 d or planned; β-blocker + verapamil/diltiazem combo ≤ 14 d or planned Septal reduction ≤ 6 mo or planned ICD placement ≤ 2 mo or planned Other significant conditions compromising safety/evaluations (per investigator) Prior cardiotoxic agents (e.g., doxorubicin)	Interventional	30 weeks of mavacamten produced marked LVOT gradient reduction, better symptoms and QoL, favorable biomarker shifts, with a manageable safety profile
NCT06549608	A Retrospective Cohort Study of Mavacamten Patient Support Program in Canada	Canada	Active, not recruiting	Age ≥ 18 years Initiated mavacamten as part of routine care through the BMS CAMZYOS Patient Support Program for symptomatic obstructive HCM Consented to use of de-identified data collected through the program	no exclusion criteria	Observational	No results posted
NCT07168655	Mavacamten in Obstructive Hypertrophic Cardiomyopathy	Japan	Active, not recruiting	Age ≥ 18; weight ≥ 35 kg Adequate windows for accurate TTE oHCM per ACCF/AHA, ESC, JCS guidelines LVEF ≥ 60% NYHA class II–III	Hypertrophy mimics Syncope or exercise-induced sustained VT ≤ 6 months Resuscitated SCA (ever) or appropriate ICD shock ≤ 6 months AF: paroxysmal with AF at screening; or persistent/permanent without ≥4 weeks anticoagulation and/or not rate-controlled ≤ 6 months Cibenzoline, disopyramide, or ranolazine ≤ 14 days or planned β-blocker + verapamil/diltiazem combo ≤ 14 days or planned Septal reduction (myectomy/ASA) ≤ 6 months or planned ICD placement ≤ 2 months or planned Other significant conditions risking safety/assessments (per investigator) Prior cardiotoxic agents (e.g., doxorubicin)	Interventional	The post-exercise LVOT gradient was measured from echocardiograms obtained at baseline and week 30 following a study-specified exercise protocol and read by the Doppler echocardiography. Baseline is defined as the last non-missing assessment prior to the first dose of the study treatment if both the time of the measurement and the time of first dose are available otherwise it is the last non-missing assessment on or prior to the first dose of the study treatment.
NCT06112743	A Study to Evaluate Mavacamten Impact on Myocardial Structure in Participants With Symptomatic Obstructive Hypertrophic Cardiomyopathy	Argentina, Australia, Canada, Switzerland, United Kingdom, United States	Active, not recruiting	oHCM diagnosed per ACCF/AHA & ESC guidelines LVOT peak gradient ≥ 30 mmHg at rest and ≥50 mmHg with Valsalva/exercise LVEF ≥ 55% at rest NYHA class II–III symptoms	Infiltrative/storage disease mimicking oHCM Obstructive CAD or prior myocardial infarction Resuscitated SCA or life-threatening ventricular arrhythmia ≤ 6 months before screening ICD or pacemaker present, or other CMR contraindication Other protocol-defined criteria apply	Interventional	No results posted
NCT07107373	A Study to Assess the Treatment of Obstructive Hypertrophic Cardiomyopathy (oHCM) With Mavacamten in the US	USA	Active, not recruiting	Age ≥ 18 years at the index date Prescription of mavacamten for the treatment of Obstructive hypertrophic cardiomyopathy (oHCM) with NYHA Class II or III ≥12 weeks of follow-up after prescription of mavacamten, except for the baseline data reporting	Data collection as part of a clinical trial during the study period Participation in a myosin inhibitor clinical trial	Observational	No results posted
NCT04766892	A Study of Mavacamten in Participants With HFpEF and Elevation of NT-proBNP With or Without Elevation of cTnT	USA, Canada	Complete	Age ≥ 50; weight > 45 kg Prior objective HF evidence (≥1): HF hospitalization + pulmonary congestion; LVEDP/PCWP ≥ 15 mmHg rest or ≥25 mmHg exercise; NT-proBNP > 400 pg/mL or BNP > 200 pg/mL; E/e′ ≥ 15 or LAVI > 34 mL/m^2^ with chronic MRA/loop Biomarker entry (meet either group): hs-cTnT ≥ 99th% and NT-proBNP > 200 (sinus)/>500 (AF); if African descent or BMI ≥ 30: >160/>400 NT-proBNP > 300 (sinus)/>750 (AF); if African descent or BMI ≥ 30: >240/>600 LVEF ≥ 60% at screening; no prior LVEF ≤ 45% Max LV wall thickness ≥ 12 mm or elevated LV mass index (>95 g/m^2^ F; >115 g/m^2^ M) High-quality TTE (±contrast) NYHA class II–III at screening	Prior HCM or infiltrative/storage hypertrophy/HFpEF (amyloidosis, Fabry, Noonan) or positive serum immunofixation Syncope ≤ 6 mo; exercise-induced sustained VT ≤ 6 mo Resuscitated SCA (any time) or appropriate ICD shock ≤ 6 mo Persistent/permanent AF without ≥ 4 wk anticoagulation and/or not rate-controlled ≤ 6 mo Planned/current: β-blocker + verapamil/diltiazem combo; disopyramide; biotin/supplements Moderate–severe AS; hemodynamically significant MS; severe MR/TR Severe COPD/other severe lung disease needing home O_2_, chronic nebulizer/oral steroids, or hospitalization ≤ 12 mo BMI ≥ 45 kg/m^2^ LV GLS 0 to −12.0 by TTE (central lab) NT-proBNP > 2000 pg/mL at screening Acute decompensated HF requiring IV diuretics/inotropes/vasodilators or LVAD ≤ 30 days	Interventional	Median age 76 years; 53% female. Biomarkers decreased: NT-proBNP −26%, hsTnT −13%, hsTnI −20% (all *p* < 0.05); values returned toward baseline 8 weeks after discontinuation. NYHA class improved in 42% of evaluable patients (10/24); echocardiographic measures of LV diastolic function also improved. Mean LVEF decreased by 3.2 percentage points (*p* = 0.005). Safety: treatment interrupted in 3/30 patients (10%) due to prespecified LVEF criteria (<50%, *n* = 2; >20% relative decrease, *n* = 1; nadir 58%); all recovered LVEF. No deaths, no LVEF < 30%; one case of worsening heart failure deemed unrelated to the study drug.
NCT07004972	A Study of Mavacamten in Adults With Obstructive Hypertrophic Cardiomyopathy in India (ROVER)	India	Recruiting	Diagnosed with obstructive hypertrophic cardiomyopathy (HCM) per ACCF/AHA and ESC guidelines Unexplained LV hypertrophy with nondilated ventricles (no hypertension, aortic stenosis, or systemic disease) Max LV wall thickness ≥ 15 mm (or ≥13 mm with positive family history) LVOT (Valsalva) peak gradient ≥ 50 mmHg (rest, Valsalva, or post-exercise) LVOT gradient ≥ 30 mmHg with Valsalva at screening TTE Adequate acoustic windows for accurate TTE NYHA Class II or III at screening Body weight > 45 kg LVEF ≥ 55% at rest on screening TTE	Infiltrative/storage disease mimicking oHCM (e.g., Fabry, amyloidosis, Noonan syndrome with LVH) Paroxysmal atrial fibrillation present on screening ECG (per investigator) Persistent/permanent AF without ≥4 weeks anticoagulation and/or not adequately rate-controlled within 6 months (note: allowed if anticoagulated and adequately rate-controlled) Syncope with exercise ≤ 6 months before screening Sustained ventricular tachyarrhythmia (>30 s) ≤ 6 months before screening Obstructive coronary artery disease (>70% epicardial stenosis) or prior myocardial infarction Other protocol-defined inclusion/exclusion criteria apply	Interventional	No results posted
NCT03496168	Extension Study of Mavacamten (MYK-461) in Adults With Symptomatic Obstructive Hypertrophic Cardiomyopathy Previously Enrolled in PIONEER	USA	Complete	Completed Study MYK-461-004. Prior participation in a non-interventional observational study is allowed. Body weight > 45 kg at Screening Has safety laboratory parameters (chemistry and hematology) within normal limits	QTcF > 500 ms or other ECG abnormality deemed a safety risk (e.g., second-degree AV block type II) Since enrollment into Study MYK-461-004: developed obstructive CAD (>70% stenosis in ≥1 artery) or known moderate/severe aortic valve stenosis Since enrollment: developed any acute or serious comorbid condition (e.g., major infection; hematologic, renal, metabolic, gastrointestinal, or endocrine dysfunction) that could pose risk or interfere with evaluations/procedures/completion Since enrollment: developed clinically significant malignant disease	Interventional	Exposure Most remained on stable doses (5/10/15 mg). 3 temporary dose reductions/interruptions for low LVEF or high levels; all recovered, maintained LVEF ≥ 50%. Safety No CV deaths or drug-related hospitalizations. One asymptomatic LVEF 47% ≈ week 180 → paused + dose reduced → normalized. AEs mostly mild/moderate (fatigue, URTI, arthralgia). Single QTc prolongation and one nonsustained VT; serious events not drug-related. Efficacy At week 180, 83% improved by ≥1 NYHA class. NT-proBNP ↓ from ~594 to 155 ng/L. LVOT gradients fell below clinically relevant thresholds in most patients. LVEF remained ≥50%; reduced SAM and MR; no septal reduction therapy needed
NCT06146660	A Study to Assess the Safety of Mavacamten in Korean Patients With Symptomatic Obstructive Hypertrophic Cardiomyopathy	USA	Recruiting	Adult participants 19 years of age or older Participants who receive mavacamten according to the approved product label Participants who sign the informed consent form	Participants who are prescribed mavacamten for therapeutic indications not approved in Korea Participants for whom mavacamten is contraindicated as clarified in Korean prescribing information approved by the Ministry of Food and Drug Safety	Observational	No results posted
NCT06551129	Real-world Patient Reported Outcomes Among Patients Treated With Camzyos	USA	Recruiting	Participants ≥ 18 years of age. Participants who are prescribed mavacamten for obstructive hypertrophic cardiomyopathy Provided informed consent to participate in the study	Previously or currently enrolled in any clinical trial of cardiac myosin inhibitors Treated with mavacamten for >7 days by the time of baseline survey completion Enrolled in any clinical trial at screening or within the prior 6 months Myocardial infarction requiring CABG within the prior 3 months Stroke or transient ischemic attack within the prior 6 months Moderate-to-severe lung disease limiting daily activities or breathing Major thoracic (lung) or cardiac surgery within the prior 6 months Scheduled major surgery in the next 3 months (e.g., joint/hip replacement, abdominal, lung, heart, eye, brain, or any surgery requiring general anesthesia and ≥1-night hospital stay) Hospitalized with an overnight stay at screening or within the prior 2 weeks	Observational	No results posted
NCT03723655	A Long-Term Safety Extension Study of Mavacamten in Adults Who Have Completed MAVERICK-HCM or EXPLORER-HCM	USA, Europe	Active, not recruiting	Completed Parent Study through EOS within 90 days of consent (>90 days may be allowed with MyoKardia Medical Monitoring approval; early discontinuations from Parent or MAVA-LTE may be considered) Body weight > 45 kg at Screening Adequate acoustic windows for accurate TTE LVEF ≥ 50% at rest on screening TTE (core lab) Safety labs (chemistry/hematology/coagulation/urinalysis) within normal limits per central lab Females: not pregnant/lactating and using a highly effective contraceptive from Screening through 90 days after last IMP dose	ECG abnormality posing safety risk (e.g., second-degree AV block type II) Syncope or sustained VT with exercise between Parent Study EOS and Screening Resuscitated sudden cardiac arrest or appropriate ICD shock for life-threatening VT/VF between EOS and Screening (ATP allowed) Disopyramide or ranolazine within 14 days prior to Screening or planned during study Acute/serious comorbidity (e.g., major infection; hematologic, renal, metabolic, GI, or endocrine dysfunction) that could risk safety or assessments Clinically significant malignant disease developed since Parent Study enrollment Unable to comply with study requirements/visits Participation in another interventional trial within 30 days prior to Screening or <5 drug half-lives (whichever longer), or current investigational device use (MAVERICK-HCM/EXPLORER-HCM allowed; non-interventional observational allowed)	Interventional	No results posted
NCT04349072	A Study to Evaluate Mavacamten in Adults With Symptomatic Obstructive HCM Who Are Eligible for Septal Reduction Therapy	america	completed	Age ≥ 18; body weight > 45 kg at screening oHCM per ACCF/AHA 2011, meeting recommendations for invasive therapy Referred/under active consideration for septal reduction therapy (SRT) within past 12 months and willing to undergo SRT LVEF ≥ 60% at screening Resting oxygen saturation ≥ 90% at screening	Persistent/permanent AF without ≥4 weeks anticoagulation and/or not adequately rate-controlled ≤6 months Prior invasive septal reduction (myectomy or ASA) BB/CCB/disopyramide dose change < 14 days before screening or anticipated change during first 16 weeks Any condition precluding upright exercise stress testing Paroxysmal/intermittent AF present at screening Prior cardiotoxic agents (e.g., doxorubicin) Any other clinically significant condition posing safety risk or interfering with study assessments/completion	interventional	At Week 16, mavacamten demonstrated: A significant reduction in SRT eligibility/need, Improved functional capacity and symptoms (NYHA, KCCQ-23), Marked decreases in cardiac stress biomarkers (NT-proBNP, troponin), A substantial reduction in LVOT gradient. While adverse events were more frequent with mavacamten—partly reflecting longer exposure—events were generally manageable with appropriate monitoring, particularly of left ventricular ejection fraction.
NCT07120776	Positron Emission Tomography to Assess the Effect of Camzyos on Ischaemia in HOCM: PEACH Trial	united kingdom	Not yet recruiting	Written informed consent Age ≥ 18 years Confirmed HOCM: unexplained LV hypertrophy with maximal wall thickness ≥ 15 mm (no uncontrolled HTN, valvular disease, or phenocopies like amyloidosis/storage disorders) and resting or provoked LVOT gradient ≥ 30 mmHg Symptoms suggestive of myocardial ischemia (e.g., chest pain, exertional dyspnea) with clinical indication for Rb-PET Eligible for mavacamten per standard clinical guidelines (routine care; not study-supplied) PET-CT previously performed for clinical reasons within the past 18 months and reported abnormal	Obstructive CAD: epicardial stenosis > 50% on invasive angiography or CTCA (angiography performed as part of routine care; a positive angiogram excludes the patient) Contraindications to mavacamten: LVEF < 55%, hypersensitivity/allergy to the drug Contraindications to rubidium PET-CT, including: Pregnancy or breastfeeding Severe claustrophobia Morbid obesity exceeding scanner capacity Any condition that, in the Investigator’s opinion, increases risk, impedes compliance, or prevents study completion	interventional	No results posted
NCT03470545	Clinical Study to Evaluate Mavacamten (MYK-461) in Adults With Symptomatic Obstructive Hypertrophic Cardiomyopathy	America-Europe	completed	Age ≥ 18; body weight ≥ 45 kg Adequate acoustic windows for accurate TTE oHCM per ACCF/AHA & ESC guidelines and both: LVEF ≥ 55%; NYHA class II–III Resting oxygen saturation ≥ 90% at screening Able to perform upright CPET with RER ≥ 1.0 at screening (central read)	Infiltrative/storage mimic of oHCM (e.g., Fabry, amyloidosis, Noonan + LVH) Syncope or exercise-induced sustained VT within 6 months Resuscitated SCA (any time) or appropriate ICD shock ≤ 6 months Paroxysmal AF present at screening Persistent/permanent AF without ≥4 weeks anticoagulation and/or not adequately rate-controlled ≤6 months Disopyramide or ranolazine ≤ 14 days or planned during study Planned/current combo: β-blocker + calcium channel blocker LVOT gradient with Valsalva < 30 mmHg at screening Septal reduction (myectomy/ASA) ≤ 6 months or planned ICD placement ≤ 2 months or planned Any other significant condition risking safety or interfering with assessments Prior cardiotoxic agents (e.g., doxorubicin)	Observational	Over 30 weeks, mavacamten: Increases positive clinical response (composite of exercise capacity and NYHA), Reduces LVOT gradient substantially, Improves exercise capacity, functional class, quality of life, and HCM symptoms, Shows a generally balanced safety profile vs placebo, with more dizziness/syncope observed—underscoring the need for clinical monitoring (e.g., symptoms, rhythm, LVEF).
NCT05489705	A Prospective Registry Study to Assess Real-world Patient Characteristics, Treatment Patterns, and Longitudinal Outcomes in Patients Receiving Mavacamten and Other Treatments for Symptomatic Obstructive Hypertrophic Cardiomyopathy (Obstructive-HCM)	Europe-USA	Recruiting	Age ≥ 18 at consent; able/willing to provide written ICF (or via LAR) US sub-study: oHCM per 2020 AHA/ACC (LV wall ≥ 15 mm, or ≥13 mm with positive family history; nondilated; not due to loading conditions) and peak LVOT gradient ≥ 30 mmHg (rest or provoked) EU sub-study: oHCM per latest ESC and AHA/ACC guidelines LVEF ≥ 55% by echo (US: within last 6 months; EU: documented by TTE) NYHA class II–IV (US)/class II–III at enrollment or within prior 6 months (EU) On routine-care therapy for oHCM (BBs, non-DHP CCBs, disopyramide, and/or mavacamten when available) or currently untreated due to intolerance/failure of prior therapy	Phenocopy disease (e.g., Fabry, amyloidosis) or LVH due to hypertension Fixed LV outflow obstruction (e.g., aortic stenosis or valve replacement) Invasive septal reduction (myectomy or ASA) within 6 months (unsuccessful procedures > 6 months may enroll) Treatment-naïve for oHCM (never treated with BBs, non-DHP CCBs, or disopyramide) US sub-study: receiving an investigational agent for oHCM (e.g., non-mavacamten myosin inhibitor) at enrollment; previously/currently in a mavacamten long-term safety study (EXPLORER-HCM, MAVA-LTE, PIONEER-OLE, VALOR-HCM, MAVERICK) EU sub-study: receiving any investigational agent or any cardiac myosin inhibitor/modulator at enrollment; previously/currently in other HCM registries (TORCH, REMY, EU-PASS); previously/currently in a mavacamten study (EXPLORER-HCM, MAVA-LTE, PIONEER-OLE, VALOR-HCM, MAVERICK, or MEMENTO) Previously treated with mavacamten	Observational	No results posted
NCT06856265	Efficacy of Mavacamten Combined With Radiofrequency Ablation in Patients With Symptomatic Obstructive Hypertrophic Cardiomyopathy	Shanghai	Active not recruiting	Age ≥ 18 years; body weight > 45 kg oHCM per ACCF/AHA, ESC, and Chinese Society of Cardiology guidelines, confirmed by core lab: Unexplained LV hypertrophy (LV wall ≥ 15 mm, or ≥13 mm with positive family history) with nondilated chambers and no secondary cause (e.g., hypertension, aortic stenosis) LVOT peak gradient ≥ 50 mmHg at rest or after Valsalva (confirmed by core lab) LVEF ≥ 55% at rest (core lab TTE) Valid Valsalva LVOT gradient measurement at screening NYHA class II–III symptoms at screening Resting O_2_ saturation ≥ 90% Able to understand and comply with study procedures and provide written informed consent	Participation in another investigational drug/device trial within 30 days or <5 half-lives Infiltrative/storage disease mimicking oHCM (e.g., Fabry, amyloidosis, Noonan + LVH) Syncope or sustained VT with exercise ≤ 6 months Resuscitated SCA (any time) or ICD shock ≤ 6 months Paroxysmal AF present at screening Persistent/permanent AF without ≥4 weeks anticoagulation and/or not rate-controlled ≤6 months (controlled/anticoagulated allowed) Previous mavacamten study participation Hypersensitivity to mavacamten components Disopyramide, cibenzoline, or ranolazine ≤ 14 days or planned β-blocker + verapamil/diltiazem combo ≤ 14 days or planned during double-blind period Recent (<14 days) or anticipated dose adjustment of BB, verapamil, or diltiazem Septal reduction (myectomy/ASA) ≤ 6 months or planned (unsuccessful >6 months allowed if eligible) ICD placement ≤ 2 months or planned QTcF > 500 ms (QRS < 120 ms) or >520 ms (QRS ≥ 120 ms), or other high-risk ECG abnormality (e.g., 2° AV block type II) CAD with >70% stenosis or prior myocardial infarction Moderate/severe aortic stenosis, constrictive pericarditis, or significant congenital heart disease Acute/serious comorbidity (e.g., infection, hematologic, renal, metabolic, GI, or endocrine dysfunction) affecting safety or assessments Inability to comply with study procedures/visits Pregnant or lactating female	interventional	No results posted
NCT06372457	COLLIGO-HCM: A Multinational Observational Study of the Real-World Effectiveness of Mavacamten Among Patients With Symptomatic Obstructive Hypertrophic Cardiomyopathy (oHCM)	USA	Active not recruiting	Source cohort: ≥1 recorded encounter with an HCM diagnosis in/after 2018 (first = index); age ≥ 18 at index; disease-specific history documented in the medical record HCM sub-cohort: participants from the source cohort with a known HCM diagnosis Mavacamten sub-cohort: participants whose first mavacamten prescription occurs after the index date	HCM sub-cohort: HCM phenocopy identified after the first HCM encounter (athlete’s heart, hypertensive heart disease, Fabry, Pompe, Danon, amyloidosis)	Observational	No results posted
NCT07150299	Myocardial Perfusion Changes Following Optimal Medical Treatment in Symptomatic Hypertrophic Cardiomyopathy	Austria	Recruiting	“Age > 18 years Willingness to provide written informed consent Diagnosis of obstructive HCM based on ESC 2023 criteria Planned CMR with myccardial perfusion for clinical purposes Receiving guideline-conform OMT Ability and willingness to undergo follow-up imaging and testing Written informed consent”	Claustrophobia or other contraindication to CMR imaging Significant coronary artery disease and/or prior stent placement or CABG surgery History of sudden cardiac arrest or sustained ventricular arrhythmia within 12 months before screening Glomerular filtration rate < 30 mL/min/m^2^ Significant hepatic impairment (≥3× upper limit of normal for transaminases, total bilirubin, or alkaline phosphatase) or hepatic cirrhosis Known allergy to contrast agent Alternative cause of hypertrophic cardiomyopathy (e.g., amyloidosis, Fabry disease) Pregnant or planning pregnancy Breastfeeding women Unwilling or unable to comply with the study protocol and procedures	Observational	No results posted
NCT07103655	The Therapeutic Value of Mavacamten in Hypertrophic Cardiomyopathy With Mid-to-Apical Left Ventricular Obstruction	China	Not yet recruiting	HCM per 2023 Chinese Guidelines, with one of: LV wall thickness ≥ 15 mm (any segment, echo or CMR), or LV wall thickness ≥ 13 mm with a pathogenic mutation or affected family history; Other causes of hypertrophy excluded. Symptomatic non-LVOT obstructive HCM (meets “a” above and ≥1 of): NYHA class II–III with dyspnea/chest pain/dizziness/palpitations/syncope, Mid-ventricular PGmax > 30 mmHg at rest or with Valsalva (echo), Apical PGmax > 30 mmHg at rest or with Valsalva (echo)	Obstructive HCM defined by LVOT-PGmax ≥ 30 mmHg at rest and with Valsalva (echo) RVOT-PGmax ≥ 16 mmHg at rest LVEF < 50% (echo) Uncontrolled primary hypertension Moderate/severe aortic stenosis and/or primary mitral valve disease with severe MR Infiltrative/storage mimics (e.g., Fabry disease, cardiac amyloidosis) Severe infection, hepatic dysfunction, renal impairment, or other serious conditions that markedly limit life expectancy	interventional	No results posted
NCT07077005	Mavacamten Enables Exercise in Hypertrophic Obstructive Cardiomyopathy	Germany		“Age ≥ 18 years of age Diagnosis of hypertrophic obstructive cardiomyopathy ≥12 weeks of unchanged dosage of mavacamten Peak left ventricular outflow tract gradient ≤ 50 mmHg at rest and during stress echocardiography Left ventricular ejection fraction ≥ 50% at study inclusion New York Heart Association classes I-II”	Syncope or sustained ventricular tachycardia within 6 months prior to inclusion Corrected QT interval (QTcF) ≥ 500 ms Paroxysmal or intermittent atrial fibrillation on screening ECG Persistent/permanent AF without ≥4 weeks of anticoagulation Prior transcoronary ablation of septal hypertrophy or surgical myectomy Ventricular tachycardia or significant ST-elevation/depression on baseline cardiopulmonary exercise test ≥Grade II valvular insufficiency or stenosis on resting echocardiography Prior implantable cardioverter-defibrillator (ICD) implantation Sudden Cardiac Death (SCD) risk score ≥ 4%	interventional	No results posted
NCT07155434	ENABLE-HCM—AI-ENabled Echocardiography With Ultrasound Beyond the Echo Lab for Better HCM Imaging and Expanded Access	USA	Recruiting	“Male and female subjects aged 18 years or over at the time of screening. Willing and able to give written informed consent. New York Heart Association (NYHA) Class I to III Eligible to receive or currently receiving CamzyosTM per product labelling.”	Emergency (non-elective) hospital admission within 24 h before study participation Inability to lie in standard TTE positions (supine/back/left decubitus) History of technically difficult echocardiogram due to body habitus (per investigator’s judgment) Body mass index (BMI) > 40 kg/m^2^ Known or suspected acute cardiac event Severe chest wall deformities per medical record or physical exam Prior pneumonectomy Anatomical variations preventing diagnostic echocardiographic imaging (e.g., situs inversus with dextrocardia, single-ventricle anatomy)	Observational	No results posted

## Data Availability

No new data were created or analyzed in this study. Data sharing is not applicable to this article.
